# *Streptococcus acidominimus* causing invasive disease in humans: a case series

**DOI:** 10.1186/1752-1947-8-57

**Published:** 2014-02-17

**Authors:** Chaoneng Wu, Buqing Liang, Yunguo Gong, Lan Zhang, Yunzeng Zou, Junbo Ge

**Affiliations:** 1 Shanghai Institute of Cardiovascular Diseases, Zhongshan Hospital, Fudan University, 180 Feng Lin Road, Shanghai 200032, China; 2 Institute of Biomedical Science, Fudan University, 138 Yixueyuan Road, Shanghai 200032, China; 3 Department of Clinical Laboratory, Zhongshan Hospital, Fudan University, Shanghai 200032, China

**Keywords:** Case series, *Streptococcus acidominimus*, Viridans group streptococci

## Abstract

**Introduction:**

*Streptococcus acidominimus* is a member of the viridans group streptococci and is rarely pathogenic in humans, making it difficult to assess its epidemiologic and clinical significance.

**Case presentation:**

We report the cases of five Han Chinese patients with invasive diseases caused by *S. acidominimus* over a one-year time frame. Three of the patients developed continuous fever after surgery, consisting of a successful elective laparoscopic cholecystectomy (case 1), a laparoscopic esophageal resection and gastroesophageal anastomosis (case 2), and a liver transplant in a patient with liver cancer (case 3). For these three patients, cultures of the purulent drainage material grew *S. acidominimus*. Case 4 concerns a 52-year-old man who developed sepsis 48 hours after hospitalization for hepatitis, liver cirrhosis and hepatitis-related glomerulonephritis. Case 5 concerns a 55-year-old woman receiving regular hemodialysis who had low-grade fever for one month. For these two patients, blood cultures grew *S. acidominimus*. An antimicrobial susceptibility test revealed that *S. acidominimus* was resistant to clindamycin and, to some degree, beta-lactam or macrolides. The *S. acidominimus* from the patient on hemodialysis was resistant to multiple antibiotics.

**Conclusion:**

*S. acidominimus* is an ever-increasing cause of disease, especially in patients who are critically ill. It is showing increased resistance to antimicrobial agents, so in patients with viridans group streptococci infections, it is necessary to identify the species to improve the clinical management of *S. acidominimus*.

## Introduction

*Streptococcus acidominimus* is a member of viridans group streptococci (VGS) [1]. Generally, *S. acidominimus* is considered to be a common bacterial pathogen in veterinary medicine. Until now, limited cases of *S. acidominimus* have been reported in humans, in pneumonia [2,3], pericarditis [4], meningitis, otitis median [5] and brain abscess [6]. Because of the poor recognition of its clinical significance in humans, identification of *S. acidominimus* as the causative species from the heterogeneous VGS has seldom been performed. Here, we report five cases of invasive diseases in a Han Chinese population caused by *S. acidominimus* over a one-year time frame.

## Case presentation

### Case 1

Our patient was a 67-year-old woman who came in for an elective operation of laparoscopic cholecystectomy. Two weeks previously, she had been diagnosed with choledocholithiasis and acute cholecystitis. Sonography and computed tomography confirmed the diagnosis. She received an emergency endoscopic retrograde cholangiopancreatography with insertion of a plastic stent (8.5F × 8.0cm) and antibiotics treatment. After that, her symptoms relieved gradually.

Her cholecystectomy was successful and after the operation she was treated with intravenous cefmetazole sodium (2g,bid). However, she then developed a continuous fever. Abdominal computed tomography one week after the surgery showed accumulated fluid in her gallbladder fossa. A catheter was used to drain the fluid and the purulent drainage material was sent for culture and cytology analysis. It grew *S. acidominimus*, identified through the Gram-positive cocci and presence of alpha hemolysis. Based on an antibiotics sensitivity test, levofloxacin (2.4g, qid) was started intravenously (Table 1). A pathology analysis revealed Rokitansky-Aschoff sinuses with chronic cholecystitis and choledocholithiasis. Our patient was discharged uneventfully 10 days later. A follow-up visit at four weeks revealed no infection.

**Table 1:** **Antimicrobial susceptibility of *Streptococcus acidominimus *** **for the patients in our case series**

**Antibiotic or antibiotic class**	**Case 1**	**Case 2**	**Case 3**	**Case 4**^**a**^	**Case 5**
Penicillin, ampicillin, and first-, second- and third-generation cephalosporins	S	S	R	R	S
Fluoroquinolones	S	S	S	S	S
Tetracycline	S	S	S	S	S
Clindamycin	R	R	R	R	R
Vancomycin	S	S	S	S	S
Linezolid	S	S	S	S	S
Azithromycin	S	S	S	R	R

^a^This patient had a mixed infection of *Enterococcus faecalis*. The susceptibility results for *E. faecalis* showed resistance to gentamycin and penicillin, but sensitivity to vancomycin, linezolid and fluoroquinolones. S, sensitivity; R, resistance.

### Case 2

A 60-year-old man underwent a chest laparoscopic esophageal resection, gastroesophageal anastomosis and jejunostomy under general anesthesia in our hospital because of esophageal squamous cell carcinoma. The operation was uneventful. After the operation, he had persistent fever. A blood test on the third postoperative day showed a white blood cell count of 15,800 cells/μL with 90.2% neutrophils. There was purulent drainage material, which showed Gram-positive cocci with alpha hemolysis. A culture grew *S. acidominimus*. Intravenous amoxicillin-sulbactam (3g, twice daily) was started for seven days (Table 1). He recovered and was discharged one month later. No recurrent infection was identified at a four-week follow-up visit.

### Case 3

A 63-year-old woman received a liver transplant after receiving a diagnosis of liver cancer one and a half years’ previously. A pathology analysis revealed cholangiocarcinoma of her whole liver, with tumor thrombus and a large area of tissue necrosis in the right branch of her hepatic portal vein. After the operation, our patient presented with continuous fever. A blood test showed normal liver and kidney functions and hepatitis virus markers were all negative. Results of a coagulation test were within the normal range. At six weeks post-operation, her white blood cell count increased to 16,200 cells/μL with 80.2% neutrophils. A chest X-ray and upper abdominal computed tomography showed a subphrenic abscess and segmental lung atelectasis. After surgical drainage, the purulent material was sent for culture, which grew *S. acidominimus*. Our patient received levofloxacin (2.4g, qid) for six days (Table 1). She recovered and was discharged at eight weeks post-operation. No recurrent infection was present at a four-week follow-up visit.

### Case 4

A 52-year-old man was admitted to our hospital because of hematuria and proteinuria of one month’s duration. He had a long history of hepatitis and decompensated liver cirrhosis with recurrent esophageal and gastric visceral bleeding. A blood test showed moderate anemia. Urine analysis showed protein, red blood cells, and a 24-hour protein measurement of 0.74g to 1.71g. His erythrocyte sedimentation rate and liver and kidney functions were all within normal ranges. He was diagnosed with hepatitis-related glomerulonephritis. Forty-eight hours after his admission, he presented with high fever, abdominal pain and vomiting. A physical examination showed abdominal tenderness and guarding, and decreased intestinal sound. An abdominal X-ray showed incomplete intestinal obstruction. A blood sample was immediately sent for culture and grew *S. acidominimus*. According to a susceptibility test, vancomycin (0.5g, bid) and levofloxacin (0.2g, bid) were initiated. One week later, he had made an uneventful clinical recovery, and was discharged after nine days of hospitalization. Follow-up at four weeks showed no recurrent infection.

### Case 5

A 55-year-old woman was transferred to our hospital because of constant fever for one month’s duration. She had been under maintenance of hemodialysis for five years due to adult polycystic kidney disease and chronic renal failure. She had moderate anemia. On admission her white blood cell count was 9000 cells/μL with 74.5% neutrophils. A chest X-ray showed nothing of note. A blood culture was performed immediately after admission. It revealed a mixed infection of *S. acidominimus* with *Enterococcus faecalis* using a BD PhoenixSpec™ Calibrator Kit analysis (Becton, Dickinson and Company, Arizona, USA).

According to a susceptibility test, she was treated with vancomycin (0.5g, four times a day) and cefminox (1g, twice a day) for seven days. Her temperature returned to normal. She was discharged after 10 days of hospitalization. A follow-up at six weeks showed no recurrent infection.

## Discussion

*S. acidominimus* is a member of VGS. Previously, the precise species of VGS has not routinely been identified. To the best of our knowledge, to date there have only been seven cases of *S. acidominimus* reported in humans (Table 2). With the advent of automated identification systems, it has become much easier to identify VGS to the species level. In the current observational study, we report five cases of infection caused by *S. acidominimus* in a Chinese population, which clearly merits special attention.

**Table 2:** **Summary of cases with invasive *Streptococcus acidominimus***

**Reference**	**Year**	**Country**	**Gender**	**Age**	**Community acquired**	**Specimen**	**Mixed infection**	**Underlying conditions**	**Infection loci**
[3]	1988	Japan	M	41	Yes	Cerebral spinal fluid	No	None	Pneumonia, pericarditis
[4]	2003	USA	M	15	Yes	Blood	No	Ventricular septal defect	Pneumonia, pericarditis
[5]	2003	Israel	M	12	Yes	Pus drainage	No	None	Otitis media (Gradenigo’s syndrome)
[7]	2004	China	M	34	Yes	Pus drainage	No	Ovarian cyst	Abdominal encapsulated effusion
[2]	2008	USA	M	55	Yes	Pleural effusion	No	Hypertension, paranoid schizophrenia	Thoracic cavity
[6]	2007	USA	M	60	Yes	Pus drainage	No	None	Brain abscess
[8]	2008	USA	F	80	Yes	Blood	No	Aortic valve replacement, diabetes mellitus, hypertension, coronary artery disease	Prosthetic valve endocarditis
			M	76	Yes	Blood	No	Non-small cell lung cancer, hypertension, coronary artery disease	Pneumonia
Present study	2011 to 2012	China	F	67	No	Pus drainage	No	Acute cholecystitis, post- endoscopic retrograde biliary drainage	Gallbladder fossa
			M	60	No	Pus drainage	No	Esophageal cancer, post operation	Peritonitis
			F	63	No	Pus drainage	No	Liver cancer, liver transplantation	Encapsulated pleural effusion
			F^a^	55	Unknown	Blood	Yes	Maintenance hemodialysis	Sepsis with no focus
			M	52	No	Blood	No	Liver cirrhosis and hepatitis-related glomerulonephritis	Sepsis with incomplete intestinal obstruction

^a^The infectious environment for this patient was undetermined because she was infected before admission, but she had long-term maintenance hemolysis. The mixed microorganism for her was *Enterococcus faecalis*. F, female; M, male.

None of our five patients had a history of pet or animal exposure or work exposure to animals or cattle. The current five cases had some clinical characteristics that were distinct from the previously reported seven cases of *S. acidominimus* (Table 2). The previous seven patients acquired the *S. acidominimus* in a community environment. Their age ranged from 12 to 80 years. Three of them (42.8%) were previously in excellent health. And no mixed infection was observed. In our five cases, the average age was 59.4 years (from 52 to 67). All had severe underlying diseases, and four of them had nosocomial infections (post-surgery). One patient was distinct in that she was on long-term maintenance hemodialysis, and developed sepsis with an undetermined infectious locus. She also had a mixed infection with *E. faecalis*. These data therefore suggest that *S. acidominimus* is causing increasingly nosocomial invasive diseases, especially in patients with severe underlying diseases.

There was also a great difference in the antimicrobial susceptibility test (Table 1). *S. acidominimus* has generally been believed to be sensitive to beta-lactam [9], as observed in the previous seven cases. However, in our five cases, there was increased resistance to beta-lactams, with two out of five being resistant. Additionally, compared to the zero resistance to macrolides in the previous cases, one of our patients was resistant. Further, the organisms in all five patients were resistant to clindamycin. The resistance of *S. acidominimus* to antibiotics warrants the accurate identification of the responsible species from the VGS to allow appropriate antibiotic treatment.

Finally, our patient on hemodialysis had a mixed infection of *S. acidominimus* and *E. faecalis*, a Gram-positive bacterium and member of the Lancefield group D streptococci [10]. Currently, *E. faecalis* is considered the second to third most common organism responsible for bacteremia in patients on chronic hemodialysis [11,12]. The infection of *S. acidominimus* mixed with *E. faecalis* was especially resistant to multiple antibiotics including beta-lactams, macrolides, and clindamycin. We were unable to find any reported cases of *S. acidominimus* infection in a patient on hemodialysis. Therefore, to the best of our knowledge, our study represents the first such report and highlights the possibility of increased resistance in the case of a mixed infection.

## Conclusions

*S. acidominimus* is an ever-increasing cause of disease, especially in patients who are critically ill. It is showing increased resistance to antimicrobial agents, so in patients with VGS infections, it is necessary to identify the species to improve the clinical management of *S. acidominimus*.

## Consent

Written informed consent was obtained from the patients for publication of this manuscript. A copy of the written consent is available for review by the Editor-in-Chief of this journal.

## Abbreviations

VGS: Viridans group streptococci.

## Competing interests

The authors declare that they have no competing interests.

## Authors’ contributions

CW, YZ and JG conceived of the study and drafted the manuscript. BL, YG and LZ read the previous relevant cases, collected the data, and performed the analysis. All authors read and approved the final manuscript.
